# Supplementary figures and images for: Genomic characterization of antimicrobial-resistance and virulence factors in Salmonella isolates obtained from pig farms in Antioquia, Colombia
Source: PLoS Negl Trop Dis. 2025 Jan 31;19(1):e0012830. doi: 10.1371/journal.pntd.0012830 (PMC11801710; doi:10.1371/journal.pntd.0012830)

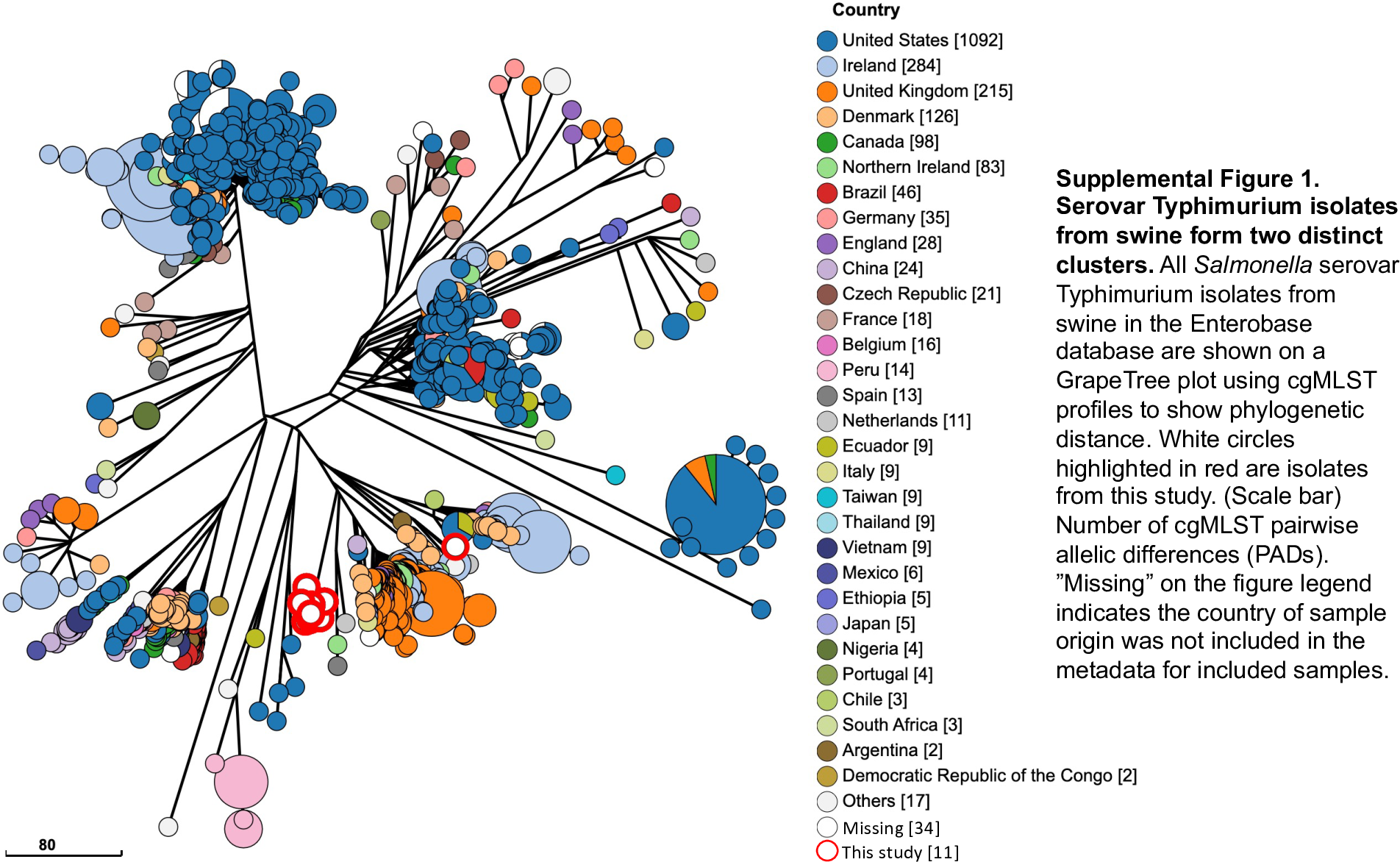

Supplement: S1 Fig — All Salmonella serovar Typhimurium isolates from swine in the Enterobase database are shown on a GrapeTree plot using cgMLST profiles to show phylogenetic distance. White circles highlighted in red are isolates from this study. (Scale bar) Number of cgMLST pairwise allelic differences (PADs).”Missing” on the figure legend indicates the country of sample origin was not included in the metadata for included samples. (TIF) [file pntd.0012830.s001.tif]
