# Supplementary material for: Genomic characterization of antimicrobial-resistance and virulence factors in Salmonella isolates obtained from pig farms in Antioquia, Colombia
Source: PLoS Negl Trop Dis. 2025 Jan 31;19(1):e0012830. doi: 10.1371/journal.pntd.0012830 (PMC11801710; doi:10.1371/journal.pntd.0012830)
Supplement: Attachment — Submitted filename: Reviewer report.pdf [file pntd.0012830.s002.pdf]

Title

Consider correcting “Genomic characterization of antimicrobial-resistant and..” (line 3) to “Genomic characterization of antimicrobial-resistance and”. The factors confer resistance, it is the Salmonella strains that are resistant.

Abstract

Background: The authors should include some comparative data from neighboring regions/countries with similar farming practices in order to contextualize their findings. In addition, they ought to specify the time period during which the samples were collected to give a clearer temporal context

Methods: While I empathize that abstracts often have word count limitations, it might be helpful to include some further details. For example, in regards to the whole genome sequencing (WGS), which platform was used? such details might be useful to a reader.
“Whole genome sequencing was performed in the Unit of Genomic of Agrosavia using an automated pipeline from the GHRU- Sanger Institute.” (line 31-33). In the methods, an Illumina MiSeq was used.

Results: The abstract mentions that 34 antimicrobial resistance genes found across 28 isolates. However, it is not clear which resistance genes were the most prevalent nor their frequencies. These resistance profiles are an important finding that could have public health/veterinary implications (e.g., co-resistance patterns).

Conclusions: The authors should consider correcting “This study showed the presence of multi-drug resistance Salmonella” (line 38) to “This study showed the presence of multi-drug resistant Salmonella”. In the conclusion, the abstract emphasizes the importance of the use of epidemiological tools, however it does not elaborate on how the findings of this study might be used. For example, do the findings from this study have any potential to advise national policy on antibiotic usage in pig farming/production systems.

Introduction

The authors should consider including some references on the possible economic impact of AMR in Columbia (or neighboring regions if the relevant literature is unavailable). [lines 93-102]

The authors could briefly comment on the current regulatory frameworks in Columbia, for example what are the national guidelines (if any) on antimicrobial usage in pig production systems? What are the most commonly used antibiotics by farmers? (this could enrich the discussion and help frame the results) and lastly any socioeconomic factors driving antimicrobial usage.

The authors could briefly outline the regional factors such as regulatory frameworks, veterinary practices, or socio-economic influences that affect antibiotic use and AMR could enrich the discussion.

Methods

Sample collection and Salmonella isolation (line 117): "A total of 28 isolates of Salmonella spp. were included…" (line 119)/" A total of 653 swine samples were collected between 2019 and 2021 from farms…." (line 211). The authors should include more detail on how the isolates were selected for the study. If the sample selection was purposive (or random) it should be stated as such. A clear inclusion and exclusion criteria should be included. Such information has far-reaching implications particularly regarding inference and generalization of the results.

Identification of presumptive bacterial isolates and antimicrobial susceptibility testing/ Genome analysis (line 138-139/line 168): The authors could consider carrying out a correlation analysis between the occurrence of antimicrobial resistance genes (genotypic resistance) and resistant phenotypes from the antimicrobial susceptibility testing (e.g., chi-square tests for categorical data).

Whole Genome Sequencing (line 150): The authors should include additional details. For example; What were the parameters used for processing the reads (e.g., quality thresholds in the analysis pipeline etc.) and assembly (e.g., trimming parameters). For each tool used it should be clearly stated which parameters were used i.e., if default settings were used it should be stated as such (e.g., BactInspector, ARIBA, and BLAST parameters). This is important in assuring transparency and reproducibility.

There's no mention of whether the generated genomic data (e.g., raw whole genome sequences) have been deposited in public databases/repositories (e.g., NCBI BioProject). Making raw sequence data publicly available is good practice as it helps facilitate data sharing, reproducibility of findings and enable further comparative analyses by other researchers.

Results

Phenotypic characterization of antimicrobial resistance (line 263): Was there any statistical testing done to determine if there were any associations between the incidence of the AMR factors and the different geographic locations? This can help give epidemiological insights on factors influencing the prevalence of antimicrobial resistance or serotype distribution in pig populations

Comparison of phenotypic and genetic resistance (line 323): The authors do not include any methodology that was used to assess this correlation. This result is thus difficult to query as there are no confidence intervals, p-values, precisions (predictive values) that could help provide insights. The significance of the reported percentages (14%, 75%, 11%) is unclear.

Discussion

More details should be included on any specific local practices, regulations that relate to antimicrobial usage in Columbia pig production systems. This can help the readers get a deeper appreciation for the authors' findings.

Incorporating statistical analysis where possible for instance on prevalence differences among serotypes and between resistance genes and phenotypic resistance could provide some quantitative support to the discussion.

The authors should specify policy recommendations drawn from the study's findings which can be used to further the discussion. For example, they can suggest actions that the stakeholders like pig farmers can take in order to be more effective in the fight against the problem of antimicrobial resistance.

References

Consider turning off URLs in the citation manager so that the references format is consistent. Some have URLs while others do not. For example "21. Wingett, S. W., & Andrews, S. (2018). Fastq screen: A tool for multi-genome mapping and quality control [version 1; referees: 3 approved, 1 approved with reservations]. F1000Research, 7(0), 1–13. https://doi.org/10.12688/f1000research.15931.1"
