# Supplementary material for: Genomic characterization of antimicrobial-resistance and virulence factors in Salmonella isolates obtained from pig farms in Antioquia, Colombia
Source: PLoS Negl Trop Dis. 2025 Jan 31;19(1):e0012830. doi: 10.1371/journal.pntd.0012830 (PMC11801710; doi:10.1371/journal.pntd.0012830)
Supplement: Attachment — Submitted filename: Rebuttal letter Plos Tropical Disease October.docx [file pntd.0012830.s004.docx]

October 6th, 2024

Dear

**Ahmed Hassan Fahal** **and Stuart Blacksell**
PLOS Neglected Tropical Diseases

Please, find the attached amendments for review comments and requests.

Thanks for your assistance

Jenny J. Chaparro-Gutierrez

1.   The CRediT author contributions  was updated.

2.    Attached separate figures.

3.The new title according to the changes suggested by the reviewers is: “Genomic characterization of antimicrobial-resistance and virulence factors in Salmonella isolates obtained from pig farms in Antioquia, Colombia (Genomic characterization of Salmonella from pig farms)”

4.     The author's summary was modified. Lines 50-62.

5.     Removed all symbols that generate copyright.

6.     Ethics committee members: Aura Sofia Vallejo, José Ignacio Calle Posada, Andrés Gomez Higuita, Anibal Vallejo Rendón, Juan Gonzalo Restrepo Salazar (Attached PDF of ethics committee approval and legal constitution of Committe).

8.     Attached figures in .TIFF format.

9.     The supporting information was added with supplemental figure 1

10.  The name of supporting figure 2 was changed to 1, as figure 1 did not exist.

11.  Table 2 was added to the manuscript.

12.  Added the information about the shapefile used in QGIS.

13. All the information from the genomes used was deposited in the National Center of Biotechnology Information (NCBI) Sequence Read Archive, with BioProject ID PRJNA1168507
