# Supplementary material for: Genomic characterization of antimicrobial-resistance and virulence factors in Salmonella isolates obtained from pig farms in Antioquia, Colombia
Source: PLoS Negl Trop Dis. 2025 Jan 31;19(1):e0012830. doi: 10.1371/journal.pntd.0012830 (PMC11801710; doi:10.1371/journal.pntd.0012830)
Supplement: Attachment — Submitted filename: Rebuttal letter PlosOne R&E.docx [file pntd.0012830.s005.docx]

November 14, 2024

Dear

**Ahmed Hassan Fahal, Stuart Blacksell and Karthiga Ravichandran**
PLOS Neglected Tropical Diseases

Please, find the attached amendments for each reviewer comments.

Thanks for your assistance

Jenny J. Chaparro-Gutierrez

**Reviewer 1:**

**Comments**

**Line 3- Correction:** Consider correcting “Genomic characterization of antimicrobial-resistant and.” (line 3) to “Genomic characterization of antimicrobial-resistance and”. The factors confer resistance, it is the Salmonella strains that are resistant. Done line 3-6. Genomic characterization of antimicrobial-resistance and virulence factors in *Salmonella* isolates obtained from pig farms in Antioquia, Colombia (Genomic characterization of *Salmonella* from pig farms).

**Abstract/Background- Correction:** The authors should include some comparative data from neighboring regions/countries with similar farming practices in order to contextualize their findings. In addition, they ought to specify the time period during which the samples were collected to give a clearer temporal context. Done. Lines 25-26 "The phrase 'Antimicrobial drug resistance in *Salmonella* strains has been associated with severe disease and greater adverse outcomes' was removed and replaced with: The presence of multidrug resistant Salmonella in pig production systems had been reported in Antioquia, Colombia Additionally, sample collection time is specified in line 30.

**Abstract/Methods- Correction:** While I empathize that abstracts often have word count limitations, it might be helpful to include some further details. For example, in regard to the whole genome sequencing (WGS), which platform was used? such details might be useful to a reader. “Whole genome sequencing was performed in the Unit of Genomic of Agrosavia using an automated pipeline from the GHRU- Sanger Institute.” (line 31-33). In the methods, an Illumina MiSeq was used. Done. Line 34. Added: employing the Illumina MiSeq platform.

**Abstract/Results- Correction:** The abstract mentions that 34 antimicrobial resistance genes were found across 28 isolates. However, it is not clear which resistance genes were the most prevalent or their frequencies. These resistance profiles are an important finding that could have implications for public health and veterinary medicine (e.g., co-resistance patterns). We consider your comment to be very important; however, due to the word count, it is not possible to include all this information. We have decided to add other important information to strengthen the abstract. Lines 34-40. Results: WGS revealed 34 ARGs among these isolates. In 25 isolates (89%) more than two ARGs were found. Genes encoding resistance were found for 10 different groups of antibiotics (beta-lactam, aminoglycosides, chloramphenicol, rifampicins, lincosamides, fluoroquinolones, tetracyclines, sulfonamides and trimethoprim). The most frequently observed MDR profile in Typhimurium isolates was AMP-CEX-CEP-CEF-EFT-CEQ-FLU-ENR-TE-FFC-SXT.

**Abstract/Conclusions- Correction:** The authors should consider correcting 'This study showed the presence of multi-drug resistance Salmonella' (line 38) to 'This study showed the presence of multi-drug resistant Salmonella.' In the conclusion, the abstract emphasizes the importance of using epidemiological tools; however, it does not elaborate on how the findings of this study might be applied. For example, do the findings from this study have any potential to inform national policy on antibiotic use in pig farming/production systems? Done. Line 40. Changed to resistant and lines 41-46. This research emphasizes the utmost importance of epidemiological tools to understand the frequency and risk factors associated with the spreading of antimicrobial resistance genes in pig farms. Additionally, it highlights the critical need for developing educational programs and public policies to help reduce the spread of antimicrobial resistance in pig production systems.

**Line 119 and 211-Correction:** A total of 28 isolates of Salmonella spp. were included (line 119). A total of 653 swine samples were collected between 2019 and 2021 from farms (line 211). The authors should provide more details on how the isolates were selected for the study. If the sample selection was purposive or random, this should be clearly stated. Clear inclusion and exclusion criteria should be provided. Such information has significant implications, particularly concerning the inference and generalization of the results. Done. 119-125. A total of 28 *Salmonella* spp. isolates were included. These were recovered from fecal samples, rectal swabs, and intestinal tissue samples from commercial pig farms in the department of Antioquia, Colombia. The samples were received at the Diagnostic Unit of the Faculty of Agrarian Sciences at the University of Antioquia, Colombia. The samples arrived at the laboratory routinely, and the inclusion period extended from January 1, 2019, to January 2021, during which they were preserved and processed.

**Lines 138-139/line 168- Correction:** The authors might consider conducting a correlation analysis between the occurrence of antimicrobial resistance genes (genotypic resistance) and the resistant phenotypes observed from antimicrobial susceptibility testing (e.g., chi-square tests for categorical data). Our team has decided to omit this section from the results. This aspect was not within the scope of the study, and we believe that conducting such analyses would require a study specifically designed with this objective in mind. Lines 330-338 in the Results section and lines 421-427 in the Discussion section have been removed.

**Line 150- Correction:** The authors should include additional details. For example, what were the parameters used for processing the reads (e.g., quality thresholds in the analysis pipeline, etc.) and for assembly (e.g., trimming parameters)? For each tool used, it should be clearly stated which parameters were applied. If default settings were used, this should be specified (e.g., parameters for BactInspector, ARIBA, and BLAST). This is important for ensuring transparency and reproducibility. Done. Lines173-195. The sequencing products were processed using an automatic pipeline from GHRU - The Centre for Genomic Pathogen Surveillance (<https://gitlab.com/cgps/ghru/pipelines>). The first pipeline is assembly and includes the read quality assessed with FastQC v0.11(21), identification of species with BactInspector v0.1.3(22) and contamination using Confidr v0.7.2(23). The sequences were trimmed and assembled using Trimommatic v0.39(24), Mash v2.2(25), Seqtk v1.3(26), FLASH v1.2.1(27), Ligther v1.1(28), and SPAdes v3.14.0.(29), in that order. The assembly's quality was determined with the software Quast v5.0.2(30). All running parameters were set as default, expect trimommatic min_length, set as 30% of raw reads size, all databases in docker containers, as described in the official pipeline page (https://gitlab.com/cgps/ghru/pipelines/dsl2/pipelines/assembly). Good quality assemblies were considered when fastqc read quality was not “FAIL” in any of the evaluated parameters, bactinspector showed species name as expected for the isolate, no contamination reported form Confindr, and assembly quality, less than 150 contigs per assembly with an N50 above 50000bp.

Prediction of AMR was performed using the second pipeline that uses ARIBA(31) against ResFinder 4.0.(32), PlasmidFinder v2.1(33), and VFDB(34) respectively (https://gitlab.com/cgps/ghru/pipelines/dsl2/pipelines/amr_prediction). For MSLT prediction MLST ARIBA software was used along with PubMLST databases(35). All the software used in this automatic pipeline were run with default parameters, their databases were in Docker containers and updated to January 2022. In silico serovar prediction, was performed by SISTR v1.1.1(36) and SeqSero2 v1.2.1.(37) packages with default parameters.

**Comment:** There is no mention of whether the generated genomic data (e.g., raw whole genome sequences) have been deposited in public databases or repositories (e.g., NCBI BioProject). Making raw sequence data publicly available is considered good practice, as it facilitates data sharing, enhances the reproducibility of findings, and enables further comparative analyses by other researchers. Done. Line 2019-222. Data availability. All WGS data supporting the findings of this study have been deposited in the National Center for Biotechnology Information (NCBI) Sequence Read Archive under BioProject ID PRJNA1168507.

**Line 263- Correction:** Was any statistical testing conducted to determine if there were associations between the incidence of AMR factors and different geographic locations? This could provide epidemiological insights into factors influencing the prevalence of antimicrobial resistance or serotype distribution in pig populations. Since the samples used in the study were received at the diagnostic laboratory as part of routine surveillance for the swine industry, we were unable to control their origin. Further, given the dissimilar quantities from each municipality, we consider that performing a correlation analysis is not feasible.

**Line 323- Correction:** The authors do not provide any methodology used to assess this correlation. As a result, the findings are difficult to evaluate, as there are no confidence intervals, p-values, or precision metrics (predictive values) to provide further insights. The significance of the reported percentages (14%, 75%, 11%) is unclear. Our team has decided to omit this section from the results. This aspect was not within the scope of the study, and we believe that conducting such analyses would require a study specifically designed with this objective in mind. The goal of this study was to assess the utility of genomics analysis for investigating Salmonella from swine as an epidemiological tool, so our results only consider the genomic and predicted data. We have edited our manuscript to make our genomics-based intent clearer, as follows: From the positive samples (n=149), we selected 28 representative isolates based on antimicrobial resistance profiles observed during phenotypic evaluation while the samples were processed in our diagnostic facility. The goal of this study was to assess the utility of genomics analysis for investigating Salmonella from swine. Line 231-235. From the positive samples (n=149), we selected 28 representative isolates based on antimicrobial resistance profiles observed during phenotypic evaluation while the samples were processed in our diagnostic facility. The goal of this study was to assess the utility of genomics analysis for investigating Salmonella from swine.

More details should be included on any specific local practices and regulations related to antimicrobial usage in Colombian pig production systems. This can help readers gain a deeper appreciation for the authors' findings. Incorporating statistical analysis where possible, such as examining prevalence differences among serotypes and between resistance genes and phenotypic resistance, could provide valuable quantitative support to the discussion. Done. Line 362-364. However, it is important to note that there are currently no regulations or action plans for antimicrobial use in pig production, nor strategies for reducing antimicrobial use in Colombia

The authors should specify policy recommendations derived from the study's findings that can be used to advance the discussion. For example, they could suggest actions that stakeholders, such as pig farmers, can take to be more effective in combating antimicrobial resistance. Done. 518-521: Furthermore, the authors underscore the imperative for the formulation of public policies aimed at regulating antibiotic use within pig production systems in Colombia. We also advocate for the establishment of educational programs designed to enhance producer awareness regarding the rational use of antibiotics.

Consider disabling URLs in the citation manager to ensure consistent formatting of references. Currently, some references include URLs while others do not. For example: WingeY, S. W., & Andrews, S. (2018). Fastq screen: A tool for multi-genome mapping and quality control [version 1; referees: 3 approved, 1 approved with reservations]. F1000Research, 7(0), 1–13. Done. All references have been modified.

**Reviewer 2:**

**Comments**

**Line 116.** The statement, “A total of 28 isolates of Salmonella spp. were included,” may be somewhat misleading, as the authors subsequently refer to “fecal samples, rectal swabs, and tissue samples.” It is unclear whether this indicates that 28 fecal, rectal swab, and tissue samples were collected or if 28 known isolates of *Salmonella spp.* were added to the samples collected. To enhance clarity, this paragraph should be revised to specify the process of sample collection. Additionally, please provide more detailed information about the “tissue” samples collected—were these blood, skin, or another type? It would also be beneficial to mention the timing of fecal sample collection: was it obtained immediately after defecation, and was it collected directly from the rectum?. Done. Lines 119-125. A total of 28 *Salmonell*a spp. isolates were included. These were recovered from fecal samples, rectal swabs, and intestinal tissue samples from commercial pig farms in the department of Antioquia, Colombia. The samples were received at the Diagnostic Unit of the Faculty of Agrarian Sciences at the University of Antioquia, Colombia. The samples arrived at the laboratory routinely, and the inclusion period extended from January 1, 2019, to January 2021, during which they were preserved and processed.

**Line 124.** Again, which tissue was collected? Done. Line 129. Gut tissue.

**Line 132.** It is worth mentioning the biochemical and serological confirmatory tests for Salmonella that were carried out on the isolates. Done. Line 138-139. These presumptive positive colonies underwent biochemical tests (TSI, LIA, citrate, and SIM) and serological tests (Check and Trace Salmonella™) for *Salmonella* confirmation.

**Line 208.** Describe the type of sampling (purposive, random) and sample size estimation calculation that was required to detect resistant genotypes, and how this was used to obtain these study samples. The 653 samples collected from 11 towns in 3 years seems few!. *Salmonella* identification testing in pigs was a new procedure in our laboratory during that period. That’s why the number of samples received was limited. Additionally, It is not mandatory to conduct this type of analysis in Colombia, so only few producers are used to collecting and sending samples to the lab for *Salmonella* Identification. The selection of the 28 samples was made randomly from the 653; however, this sampling was intentional, as we had limited resources for conducting phenotypic tests and whole genome sequencing. Done. Line 229-241. A total of 653 swine samples were collected between 2019 and 2021 from farms across various municipalities in Antioquia, with 22.8% (149/653) testing positive for Salmonella. From the positive samples (n=149), we selected 28 representative isolates based on antimicrobial resistance profiles observed during phenotypic evaluation while the samples were processed in our diagnostic facility. The goal of this study was to assess the utility of genomics analysis for investigating Salmonella from swine. Of the 28 samples, 8 were gut tissue, 15 were fecal samples, and 5 were rectal swabs. The distribution of these isolates across towns was as follows: Don Matías (n=7), Amalfi (n=3), Angelópolis (n=4), Caldas (n=2), Entrerríos (n=1), Medellín (n=1), San Pedro de los Milagros (n=1), Santa Rosa de Osos (n=6), Santo Domingo (n=1), Támesis (n=1), and Valparaíso (n=1) (see Figure 1). Whole genome sequencing analysis identified three serotypes: Typhimurium (n=10, 36%), Altona (n=3, 11%), and I 4,[5],12:i:- (n=15, 53%).

Additionally, a section on study limitations has been included, clarifying that economic factors influenced the selection of the samples. Line 499-509. The limitation of resources in this study precluded the phenotypic and genome sequencing analysis of all *Salmonella* isolates. The authors believe that employing such technology would be of significant value for molecular epidemiology studies. It would enhance our understanding of the dynamics of antimicrobial resistance in *Salmonella* isolates in Antioquia, Colombia. Further, complete genome sequencing could provide deeper insights into the genetic mechanisms driving resistance, facilitate the identification of novel resistance genes, and support the development of more targeted and effective control strategies. Addressing this limitation in future research could greatly contribute to a more nuanced understanding of resistance patterns and inform public health and policy interventions.

**Comment:** The 28 representative samples, it is worth mentioning the sample source as indicated under the methodology sample collection section, for instance how many of these 28 isolates were collected from fecal, rectal swab or tissue. Done. Line 229-241. A total of 653 swine samples were collected between 2019 and 2021 from farms across various municipalities in Antioquia, with 22.8% (149/653) testing positive for Salmonella. From the positive samples (n=149), we selected 28 representative isolates based on antimicrobial resistance profiles observed during phenotypic evaluation while the samples were processed in our diagnostic facility. The goal of this study was to assess the utility of genomics analysis for investigating Salmonella from swine. Of the 28 samples, 8 were gut tissue, 15 were fecal samples, and 5 were rectal swabs. The distribution of these isolates across towns was as follows: Don Matías (n=7), Amalfi (n=3), Angelópolis (n=4), Caldas (n=2), Entrerríos (n=1), Medellín (n=1), San Pedro de los Milagros (n=1), Santa Rosa de Osos (n=6), Santo Domingo (n=1), Támesis (n=1), and Valparaíso (n=1) (see Figure 1). Whole genome sequencing analysis identified three serotypes: Typhimurium (n=10, 36%), Altona (n=3, 11%), and I 4,[5],12:i:- (n=15, 53%).

**Line 219.** The context under this subsection involved comparison of the Typimurium isolates from this study with global isolates from Enterobase database. I suggest the subsection heading is changed to “comparison with global serovar Typhimurium isolates”. Done. Line 243. Changed to comparison with global serovar Typhimurium isolates.

**Line 270.** Change subtitle to “identification of antimicrobial resistance genes”. Done. Line 300. Changed to identification of antimicrobial resistance genes.

**Line 322.** When comparing phenotypic and genotypic resistant isolates, the authors mention correlation analysis however there is no correlation coefficients nor r-squared values given to support the claims. Support the term “discrepancy” with the correlation coefficient values for the comparison. Our team has decided to omit this section from the results. This aspect was not within the scope of the study, and we believe that conducting such analyses would require a study specifically designed with this objective in mind. Lines 330-338 in the Results section and lines 421-427 in the Discussion section have been removed.

**Editor comments**

1) **Please upload the separate figure files in numerical order.** Done. Attached separate figures.
3) **Please ensure that the Title in your manuscript file and the Title provided in your online submission form are the same.** The new title according to the changes suggested by the reviewers is: Genomic characterization of antimicrobial-resistance and virulence factors in Salmonella isolates obtained from pig farms in Antioquia, Colombia (Genomic characterization of Salmonella from pig farms)
4) We note that you have included "Author Summary" in the Manuscript file. Please note that this SHOULD BE 150-2OO WORDS . The aim should be to make your findings accessible to a wide audience that includes both scientists and non-scientists. Sample summaries can be found on our website under Submission Guidelines:
   <https://journals.plos.org/plosntds/s/submission-guidelines#loc-parts-of-a-submission>. Done. Lines 50-62: Recent research has raised alarms about antimicrobial resistance in Salmonella found in pigs from farms in Antioquia, Colombia. We used whole-genome sequencing to identify 34 resistance genes in 28 Salmonella isolates, revealing that 89% of these isolates carried more than two resistance genes, affecting various antibiotics. This situation poses significant risks, as multidrug-resistant Salmonella in pigs could directly or indirectly impact human health and lead to potential economic losses in pig production.Our findings highlight the urgent need to implement better management practices on farms and responsible antibiotic use in pig production. Properly addressing these issues is essential to ensure food safety and protect public health. Continuous surveillance and effective control measures are crucial to combat this growing problem. By prioritizing these actions, we can help mitigate the risks associated with antimicrobial resistance and safeguard both livestock and consumers.
6) We do not publish any copyright or trademark symbols that usually accompany proprietary names, eg ©,  ®, or TM  (e.g. next to drug or reagent names). Therefore please remove all instances of trademark/copyright symbols throughout the text, including:  Salmonella™ on line no 138. PureLink™ on line no 156, NanoDrop® on line no 158, NEBNext® Ultra™ on line no 160, and Compact® on line no 147. Done. Removed all symbols that generate copyright.
7) Thank you for stating"The Ethics Committee for Animal Experimentation (CEEA) of the Universidad de Antioquia granted the ethical endorsement to perform this study according to minute No. 140 of June 1, 2019. An error was made in the ethics committee, and the ethics committee document is provided, including the name of the institution and the date. Lines 114-116: The Ethics Committee for Animal Experimentation (CEEA) of the Universidad de Antioquia granted the ethical endorsement to perform this study according to minute No. 141 of August 3, 2021.
8) 8) Please upload all main figures as separate Figure files in .tif or .eps format. For more information about how to convert and format your figure files please see our guidelines.  Attached figures in .TIF format.
9) We note that you have uploaded your " Supplemental Figure2" as Figure type. Please amend the file type to 'Supporting Information'. Please ensure that each Supporting Information file has a legend listed in the manuscript before or after the references list. Done.  The supporting information was added with supplemental figure 1.
10) We have noticed that you have not cited and not uploaded Supplemental Figure1 .Please upload it as separate files with the item type 'Supporting Information'. Done.  The name of supporting figure 2 was changed to 1, as figure 1 did not exist.
11) Tables should not be uploaded as individual files. Please remove these files and include the Tables in your manuscript file as editable, cell-based objects. Done. Table 2 was added to the manuscript.
12) Figure 1. Please (a) provide a direct link to the base layer of the map (i.e., the country or region border shape) and ensure this is also included in the figure legend; and (b) provide a link to the terms of use / license information for the base layer image or shapefile. We cannot publish proprietary or copyrighted maps (e.g. Google Maps, Mapquest) and the terms of use for your map base layer must be compatible with our CC BY 4.0 license. Done. Figure 1. The geographical map depicts the location of Salmonella isolates in the different municipalities of Antioquia, Colombia. The base map shapefile used in Qgis 3.34 was sourced from the publicly database Colombia en Mapas ([https://www.colombiaenmapas.gov.co](https://www.colombiaenmapas.gov.co/))
13) Please update your Data Availability statement in the submission form accordingly. Done. Line 2019-222. Data availability. All WGS data supporting the findings of this study have been deposited in the National Center for Biotechnology Information (NCBI) Sequence Read Archive under BioProject ID PRJNA1168507.
14) Please amend your detailed Financial Disclosure statement. This is published with the article, therefore should be completed in full sentences and contain the exact wording you wish to be published. Done. Lines 535-538. This study was supported by the Sustainability Strategy of the CIBAV Research, School of Veterinary Medicine, Faculty of Agriculture Science, University of Antioquia (UdeA), Medellín, Colombia, and the Global Health Research Unit for the Genomic Surveillance of Antimicrobial Resistance-Colombia
